# Synergetic Functional Nanocomposites Enhance Immunotherapy in Solid Tumors by Remodeling the Immunoenvironment

**DOI:** 10.1002/advs.201802012

**Published:** 2019-02-16

**Authors:** Linnan Yang, Jing Sun, Qiang Liu, Rongrong Zhu, Qiannan Yang, Jiahui Hua, Longpo Zheng, Kun Li, Shilong Wang, Ang Li

**Affiliations:** ^1^ Research Center for Translational Medicine at East Hospital Shanghai First Maternity and Infant Health Hospital School of Life Science and Technology Tongji University Shanghai 200092 P. R. China; ^2^ Shanghai Tenth People's Hospital School of Medicine Tongji University Shanghai 200092 P. R. China

**Keywords:** immunosuppressive tumor microenvironments, immunotherapy, layered double hydroxides, microRNA, nanoparticles

## Abstract

Checkpoint blockade immunotherapy has demonstrated significant clinical success in various malignant tumors. However, the therapeutic response is limited due to the immunosuppressive tumor microenvironment (ITM). In this study, a functional nanomaterial, layered double hydroxides (LDHs), carrying specific functional miR155 is developed to modulate ITM by synergistically repolarizing tumor associated macrophages (TAMs) to M1 subtype. LDH nanoparticles loaded with miR155 (LDH@155) exhibit superior ability in cellular uptake by murine macrophages, miR escape into the cytoplasm and TAMs specific delivery when introtumoral administration. Meanwhile, upon exposure to LDH@155, TAMs are significantly skewed to M1 subtype, which markedly inhibits myeloid‐derived suppressor cells (MDSCs) formation and stimulates T‐lymphocytes to secrete more interferon‐γ (IFN‐γ) cytokines in vitro. Introtumoral administration of LDH@155 reduces the percentage of TAMs and MDSCs in the tumor and elevates CD4^+^ and CD8^+^ T cell infiltration and activation, which can promote therapeutic efficiency of α‐PD‐1 antibody immunotherapy. Furthermore, it is found that LDH@155 significantly decreases the expression level of phosphorylated STAT3 and ERK1/2 and activates NF‐κB expression in TAMs, indicating that the STAT3, ERK1/2, and NF‐κB signaling pathways may involve in LDH@155‐induced macrophage polarization. Overall, the results suggest that LDH@155 nanoparticles may, in the future, function as a promising agent for cancer combinational immunotherapy.

## 1 Introduction

Cancer immunotherapy has emerged as a promising strategy in recent years for various cancers.^1^, ^2^, ^3^ However, combating solid tumors still remains an enormous challenge because of the immunosuppressive tumor microenvironment (ITM), which hinders antitumor immunity and promotes cancer progression.^4^ Effective antitumor immunity in tumor environment is frequently impeded by immunosuppressive cells which are mediated by a series of tumor‐derived factors: (1) tumor cell expression of ligands that mediate T cell dysfunction,^5^, ^6^ (2) tumor‐induced antitumor M1 macrophages repolarization to tumorigenic M2 macrophages, etc.^7^, ^8^, ^9^

Numerous findings suggest that tumors recruit a constant influx of M2 subtype tumor associated macrophages (TAMs) in solid tumor^10^ and a high TAM density positively correlates with poor prognosis in many human cancers.^11^, ^12^ M2 subtype TAMs have a crucial role in regulating every aspect of tumor development, namely growth, survival and metastasis of tumor cells, angiogenesis, and immunosuppression.^8^, ^13^ Therefore, repolarizing M2 subtype TAMs to M1 might offer novel targets for therapeutic intervention aimed at depriving tumor growth.^14^, ^15^, ^16^ Currently, these therapeutic approaches targeting TAMs included: (1) inducing cell apoptosis directly,^17^ (2) reducing TAMs infiltration in tumor area through interfering the effects of relevant recruitment factor,^18^, ^19^ and (3) skewing TAMs to M1 subtype and remodel a tumoricidal immune environment.^20^

MicroRNAs, a group of small noncoding RNAs, show growing effects in regulating gene expression through binding to the 3′ untranslated region of target mRNAs.^21^ MicroRNAs have been considered as effective regulators of immune cell function.^22^, ^23^ MicroRNA‐155(miR155), upregulated in both myeloid and lymphoid cells, is one of the most excellent miRNA related to inflammation.^24^, ^25^ MiR‐155 shows promising role for development and function in both innate and adaptive immune cells, such as enhancing effector CD8^+^ T cell responses and modulating tumor‐infiltrating dendritic cells (DCs) and TAMs to potentiate cancer immunotherapies.^26^, ^27^, ^28^ Hence, miR155 might be a potential gene drug to overcome immunosuppression within tumor environment so as to inhibit tumor growth ultimately. However, these free gene drugs could not realize effectively endosomes/lysosomes escape, generating weak immune response in tumor.^29^

With the development of nanotechnology, nanomaterials have emerged as a promising approach in the cancer immunotherapy.^30^, ^31^, ^32^, ^33^, ^34^ Compared to free gene‐drug, the delivery system loading miR has a variety of benefits, for example, constant release of miR from nanoparticles at the injected site for a prolonged period, protection of miR from enzymatic degradation and enhancing cytosolic delivery of miR.^35^, ^36^ Layered double hydroxides (LDHs), also known as hydrotalcite‐like compounds, were usually used as nanovectors.^37^, ^38^ In our previous study, LDH nanoparticles show superior capability in upregulating proinflammatory cytokines (TNF‐α, IL‐12) and costimulatory molecules (CD40, CD80, CD86, and MHC class II) in marrow‐derived dendritic cells.^39^

Considering that macrophages originate from the same myeloid‐derived cells as dendritic cells and the merit of miR155 and LDH nanoparticles in inducing production of proinflammatory cytokines in myeloid cells, it seems plausible that LDH NPs‐loaded miR155 as a nanocomposite called LDH@155 might be strongly reversing ITM by repolarizing TAMs.

In this study, we sought to determine the effect of LDH@155 in remolding ITM in TC‐1 mouse tumor model. So the aim of this work was to (1) certify the efficient endosomal escape of LDH@155 in order to realize cytoplasmic release, (2) evaluate the ability of LDH@155 to inhibit tumor growth and remodel tumor immune environment by repolarization of TAMs, (3) confirm the LDH@155 could enhance therapeutic effects of immunocheckpoint inhibitor therapy in combination with programmed cell death‐1 antibody (α‐PD‐1) antibody, (4) explore the molecular mechanism involved in repolarization of macrophage by LDH@155. Therefore, we have verified that this nanocomposite could remold ITM and act as a promising agent for cancer combinational immunotherapy.

## 2 Results and Discussion

### 2.1 Characterization of LDH@miR NPs

**Figure 1:**
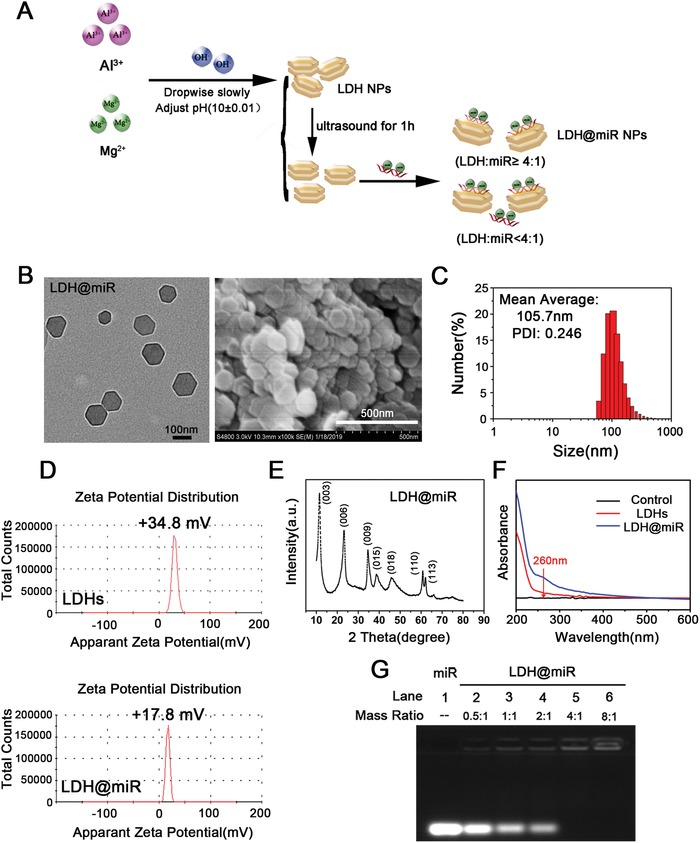
Preparation and characterization of LDH@miR NPs. A) Schematic representation of LDH@miR preparation. B) TEM image (left) and SEM image (right) of LDH@miR. (TEM image: bar = 100 nm; SEM image: bar = 500 nm). C) Particle size distribution of LDH@miR determined by DLS. D) Zeta potential of LDHs and LDH@miR (LDH:miR = 20:1, w/w) at 25 °C. E) XRD analysis of LDH@miR. F) UV absorption spectrum of LDH@miR and LDHs. G) miR loading capacity detected by agarose gel electrophoresis.

We first synthesized LDHs through coprecipitation method. Then cationic LDHs were mixed with miR at 4 °C for 30 min to obtain LDH@miR (**Figure** **1**A). Transmission electron microscope (TEM) image and scanning electron microscope (SEM) image showed LDH@miR had a narrow distribution and dispersed uniformly morphology based on regular hexagon (Figure 1B). LDH@miR had a mean hydrodynamic diameter about 105 nm with polydispersity index at 0.246 which indicated well uniformity of nanoparticles (Figure 1C). Compared to pristine LDHs, LDH@miR appeared a zeta potential at +17.8 mV, which not only indicated successful loading of miR but also exhibited a moderate potential to be internalized easily by cell membranes which presented negative charges (Figure 1D). The X‐ray diffraction (XRD) analysis of prepared LDH@miR showed strong reflections of (003) and (006), revealing the formation of crystalline layered structure (Figure 1E). In Figure 1F, UV spectrum exhibited obvious absorption crest at 260 nm of LDH@miR, because of existing of miR on LDHs. Then we investigated the saturation of the miR binding to LDHs through the agarose gel retardation assay. As shown in Figure 1G, free miR was migrated to the bottom and presented a bright band. Instead, with the mass ratio enhanced gradually, the majority of miR intergrated to LDHs were delayed in the well due to electrostatic interactions while unabsorbed miR was slowly migrated to the bottom. LDH NPs can effectively bind with miR at quite low mass ratio of 4:1 (LDH:miR).

### 2.2 Cellular Uptake and Isolation, Acid Release, TAM‐Targeting, and Metabolism of LDH@155 Nanoparticles In Vitro and In Vivo

**Figure 2:**
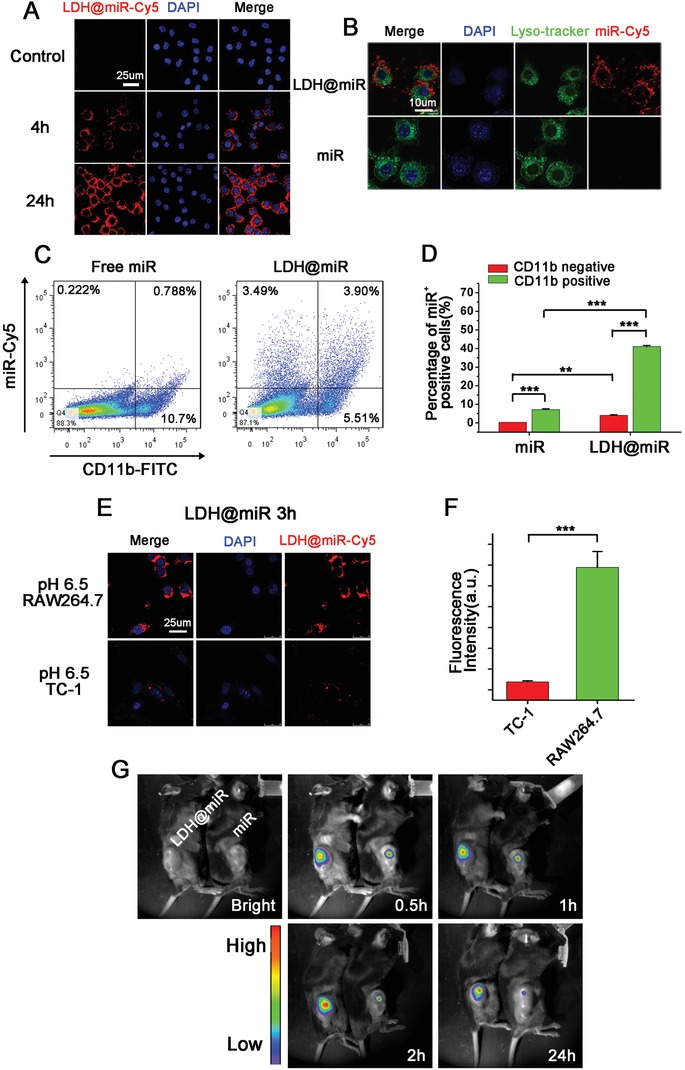
Cellular uptake and isolation, TAM‐targeting and metabolism of LDH@155 nanoparticles in vitro and in vivo. A) Fluorescence confocal images of LDH@miR‐Cy5 uptake by RAW264.7. Bar = 25 µm. B) Intracellular colocalization of LDH@miR‐Cy5 and miR‐Cy5 in RAW264.7 after incubation for 3 h, and labeled with DAPI (blue) and Lyso‐tracker (green) to distinguish cell nuclei and lysosome, respectively. Bar = 10 µm. C) Tumor‐bearing C57BL/6J mice were i.t. injected with free miR‐Cy5 or LDH@miR‐Cy5. The uptake of miR by TAMs (CD11b^+^) of TC‐1 tumors was detected at 48 h using flow cytometry. D) Quantitative percentage of miR^+^ cells in CD11b positive and negative cells in TC‐1 tumors treated by LDH@miR or free miR, respectively. E) Fluorescence confocal images of LDH@miR‐Cy5 uptake by RAW264.7 and TC‐1 at pH 6.5 for 3 h, respectively. Bar = 25 µm. F) Quantitative fluorescence intensity of miR‐Cy5 in TC‐1 and RAW264.7, respectively. G) TC‐1 tumor‐bearing mice were i.t. injected with free miR‐Cy5 or LDH@miR‐Cy5 for in vivo imaging at different time points. Date are presented as mean + s.d. Statistical significance was calculated by Student's *t*‐test and one‐way ANOVA, **p < 0.01; ***p < 0.001.

In order to evaluate transfection capacity of LDH@miR, mouse RAW 264.7 macrophages were incubated with LDH@miR‐Cy5 for 4 h and 24 h. As shown in **Figure** **2**A, at 4 h, clear fluorescence of miR‐Cy5 was displayed surrounding cell nucleus. And the fluorescence intensity was enhanced clearly at 24 h, indicating LDH@miR were swallowed by macrophages with time dependent. Meanwhile, we also found that most of LDH@miR were distributed in cytoplasm rather than lysosome due to proton sponge effect which caused endosome/lysosomal escape of miR.^40^ However, free miR was difficult to enter cells and easy to be captured by lysosome, which is according with previous report.^41^ (Figure 2B) Hence, LDH@miR could deliver miR efficiently to cytoplasm and incorporate into RISC to exert function which could enhance bioavailability of miR obviously.

In addition, pH‐sensitive capacity of nanoparticles is important for miR‐based nanotherapeutics. So, we evaluated whether it could realize effective release in simulated physiological conditions via agarose gel retardation assay firstly. As shown in Figure S1A of the Supporting Information, LDH@miR was treated by acid activation under different pH values for 1 h. The bands turned from weak to bright with the pH value reducing gradually and got similar release compared to control at pH 4.5–5.5. Furthermore, the release amount of miR was explored with time extending at pH 5.5. The similar result was showed in Figure S1B of the Supporting Information. These acid‐sensitive release abilities of LDH@miR could realize no miR leakage at physiological condition (pH 7.4) and slight release at extracellular environment of tumor (pH 6.5). However, once uptaken by macrophages, miR could release from nanoparticles under the acid environment of endosome/lysosome (pH 4.5–5.5).

Next, we further investigated whether phagocytosis difference existed between weak acid and normal physiological state in vitro, in consideration of the weak acid condition of tumor environment (pH 6.5). As shown in Figure S2A of the Supporting Information, at acid atmosphere (pH 6.5), LDH@miR uptaken by macrophages were enhanced clearly compared to neutrally condition (pH 7.4) at 1 h. The status was remained up to 3 h (Figure S2B, Supporting Information). This consequence indicated LDH@miR could be swallowed faster by macrophages in tumor microenvironment compared to normal physical condition.

Furthermore, acid‐sensitive phagocytosis by macrophages was investigated in tumor environment of TC‐1 model in vivo. As shown in Figure 2C,D, among CD11b^+^ cells which mainly TAMs, in LDH@miR group, about 41.44% were miR positive cells. However, only 6.86% miR^+^ cells were entered into CD11b negative cells. Meanwhile, about 7.15% was CD11b^+^miR^+^ cells in free miR group. These result suggested LDH@miR could not only facilitate macrophage‐targeted delivery but accelerate miR uptake by macrophages. To confirm whether LDH@miR selectively entered into macrophages, we evaluated phagocytosis difference between TC‐1 tumor cells and RAW264.7 macrophages at pH 6.5 to simulate tumor micro‐environment. As the results in Figure 2E,F, LDH@miR demonstrated strong fluorescent signals in RAW264.7 cells after incubation for 3h. Meanwhile, negligible signals were found in TC‐1 cells compared to RAW267.4 cells dealt with the same process. These results suggested that LDH@miR could be easier swallowed by macrophages compared to tumor cells either in vitro or in vivo, which would achieve better effects in TAM repolarization to realize tumor recession ultimately. Several possible reasons may contribute to account for this result, such as (1) some receptors on macrophages may be specific bind by LDH@miR,^42^, ^43^ (2) moderate size of NPs was also contributed to endocytosis of macrophages,^44^ and 3) macrophage have stronger phagocytosis ability than other cells.

We further evaluated the retention time of LDH@miR‐Cy5 in tumor by real‐time monitoring via in vivo imaging system. As shown in Figure 2G, at 0.5 h free miR‐Cy5 had a stronger fluorescence intensity than LDH@miR‐Cy5. But with the extension of time to 2 h, the fluorescent signal of LDH@miR got more and more brighten while free miR got a bit recession. When we monitored the signal until 24 h, the signal of LDH@miR‐Cy5 still remained strong but free miR‐Cy5 was almost invisible. This result may due to miR‐Cy5 was encapsulated by LDH NPs, so the signal was a bit weaker at the beginning. But with the time extension, miR‐Cy5 was released from LDH@miR‐Cy5 so as to emerge more strong fluorescence than free miR‐Cy5. And strong fluorescence was found at 24 h of LDH@miR, suggesting LDH@miR could improve bioavailability of miR obviously to realize more enduring effect in vivo.

### 2.3 LDH@155 Repolarized TAMs into Antitumor M1 Macrophages In Vitro

**Figure 3:**
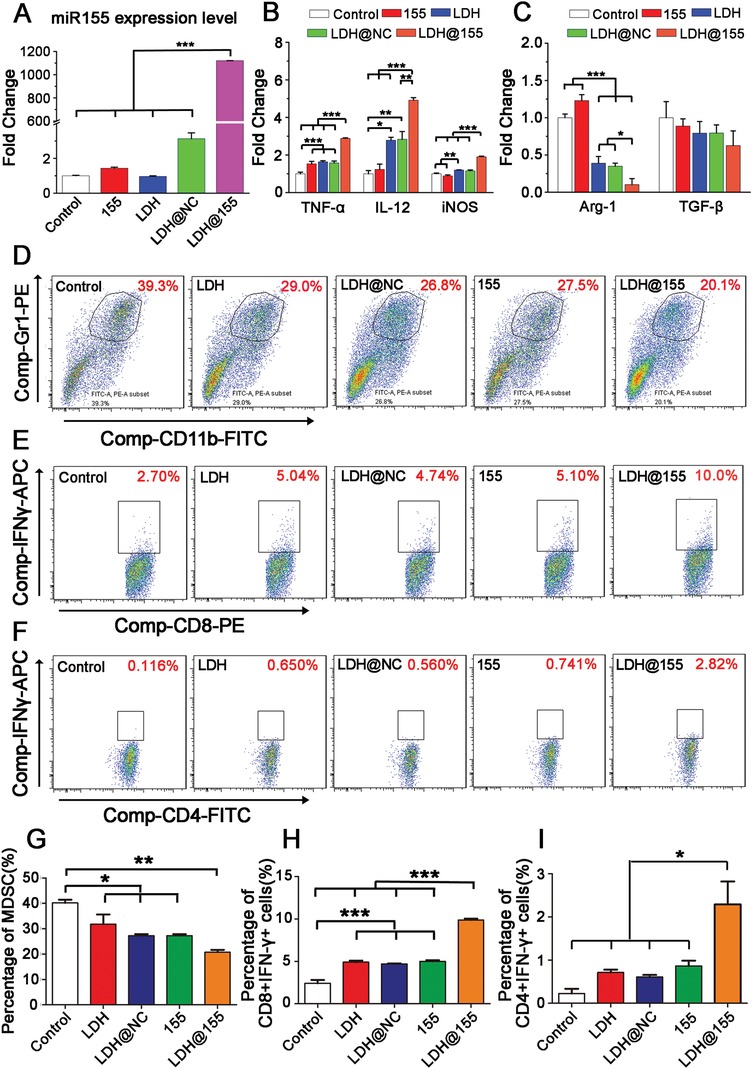
LDH@155‐induced macrophage state switch in vitro. Macrophage extracted from abdomen of C57BL/6J female mice were induced to TAMs and then treated with LDH NPs, LDH@NC, free miR155, and LDH@155. A) The expression of miR155 was detected by qRT‐PCR and normalized to U6 at 48 h. The mRNA expression level of B) M1 macrophage markers (TNF‐α, IL‐12, iNOS) and C) M2 macrophage markers (Arg‐1, TGF‐β) in TAMs at 48 h. D) Representative flow cytometry images and G) percentage analysis of MDSCs formation after incubating with TAMs treated with different miR‐NPs. E) Representative flow cytometry images and H) percentage analysis of CD8^+^ T cells activation after incubating with TAMs supernatant treated with different miR‐NPs. F) Representative flow cytometry images and I) percentage analysis of CD4^+^ T cells activation after incubating with TAMs supernatant treated with different miR‐NPs. Date are presented as mean + s.d. Statistical significance was calculated using one‐way ANOVA. *p < 0.05; **p < 0.01; ***p < 0.001.

Given that both LDH and miR155 have potential ability to promote M1 subtype repolarization, we next evaluate whether LDH@155 has synergetic effect on repolarize macrophage to M1 subtype. Macrophages were extracted from abdomen of C57BL/6J female mice and induced by macrophage colony‐stimulating factor (M‐SCF) cytokines and TC‐1 cell culture supernatant to obtain TAMs (Figure S3, Supporting Information). Cytotoxicity of LDH@155, free miR155, and equivalent LDH NPs on TAMs were evaluated using MTT assays and no obvious cytotoxicity were found in all treated groups even up to 48 h (Figure S4, Supporting Information). Then TAMs were treated with LDHs, LDH@NC (negative control miRNA), free miR155, and LDH@155 followed by detection miR155 expression and M1, M2 marker. As shown in **Figure** **3**A, LDH@155 could enhance the expression of miR155 greatly about 1100‐fold in TAMs compared with control. Meanwhile, as we expected, miR155, LDH, and LDH@NC all failed to promote miR155 expression in TAMs. Furthermore, LDH@155 could increase M1 macrophage markers and decrease M2 macrophage markers prominently (Figure 3B,C), such as elevating iNOS, IL‐12, TNF‐α by about twofold, threefold, fivefold and reducing Arg‐1, TGF‐β by 85% and 45%, respectively, compared with control group. Free miR155 had no obvious effects on either M1 markers or M2 markers due to defection of transfection capacity. Though LDH or LDH@NC treatment increased the expression level of M1 markers to some degree, their influences were weaker than LDH@155.

Considering TAMs have the ability to influence the formation of myeloid‐derived suppressor cells (MDSCs), a class of bone‐marrow derived suppressor cells, and activation of T cells. We further evaluated the effect of LDH@155 on TAM polarization through observation of formation of MDSCs and activation of T cells when coculturing with LDH@155 treated TAMs. As shown in Figure 3D,G, TAMs reeducated by LDH@155 decreased the forming percentage of MDSCs by 50%. Meanwhile, we found that percentage of activated CD8^+^ T cells (Figure 3E,H) and CD4^+^ T cells (Figure 3F,I) were elevated up to about four and ten times in TAMs treated by LDH@155 group compared to control group, respectively. LDH, LDH@NC or free miR155 alone treatment just slightly reduced MDSC formation and promoted CD8^+^ and CD4^+^ T cell activation. These results indicated LDH@155 could repolarize protumorial M2 macrophages to antitumor M1 macrophages more effectively than LDH, LDH@NC or free miR155 treatment alone. LDH@155 significantly inhibited production of anti‐inflammatory cytokines such as TGF‐β, IL‐10, and promoted IL‐12 secretion to facilitate CD8^+^ and CD4^+^ T cell activation in order to realize tumor recession ultimately. So, LDH@155 may serve as effective nanodrug to remodel tumor immune environment in solid tumor.

### 2.4 LDH@155 Could Remold Tumor Immunosuppressive Environment In Vivo

**Figure 4:**
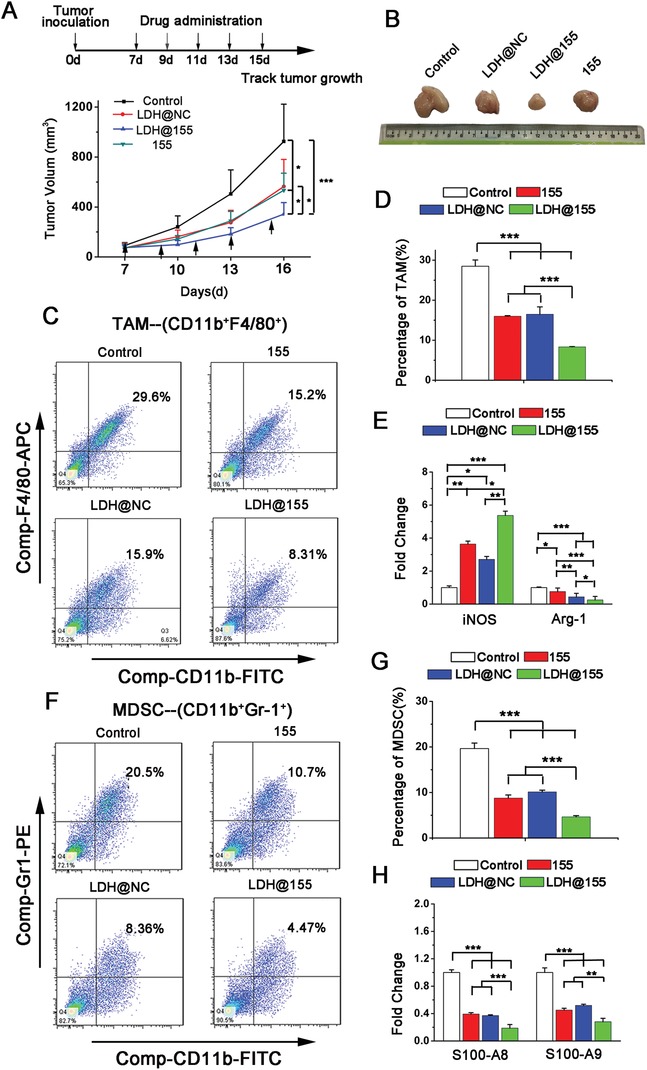
Tumor growth inhibition and immunosuppressive environment remolding in TC‐1 models by LDH@155. TC‐1 tumor‐bearing mice (*n* = 5) were i.t. injected with PBS, LDH@155 or LDH@NC, free miR155 every 2 days from day 7 after tumor implantation. A) Tumor growth was monitored every 3 days. B) Side‐by‐side comparison of tumors on day 17 after tumor extracted from mice body. C) Representative flow cytometry images of tumor‐associated macrophages (TAMs) from TC‐1 tumors. D) Percentage of TAMs within suspension cells from the TC‐1 tumors after different treatments. E) The mRNA expression level of iNOS (M1 marker) and Arg‐1(M2 marker) in TAMs detected by qRT‐PCR. F) Representative flow cytometry images of myeloid‐derived suppressor cells (MDSCs) from TC‐1 tumors. G) Percentage of MDSCs within suspension cells from the TC‐1 tumors after different treatments. H) The mRNA expression level of S100A8, S100A9 in MDSCs detected by qRT‐PCR. Date are presented as mean + s.d. Statistical significance was calculated by using one‐way ANOVA. *p < 0.05; **p < 0.01; ***p < 0.001.

In order to evaluate the effect of LDH@155 on TAMs and remolding ITM in vivo, TC‐1 tumor‐bearing C57BL/6J mice were treated with PBS, LDH LDH@NC, LDH@155, and free miR155 on day 7 after tumor implantation for four times every 2 days via i.t injection. As shown in **Figure** **4**A,B, LDH@155 exhibited effectively tumor‐growth inhibition up to 65% compared to control group. Meanwhile, LDH@NC and LDH also had moderate effects in tumor growth inhibition, which was consistent with the finding that LDH NPs also had the ability to skew the TAM to M1 subtype in vitro.

**Figure 5:**
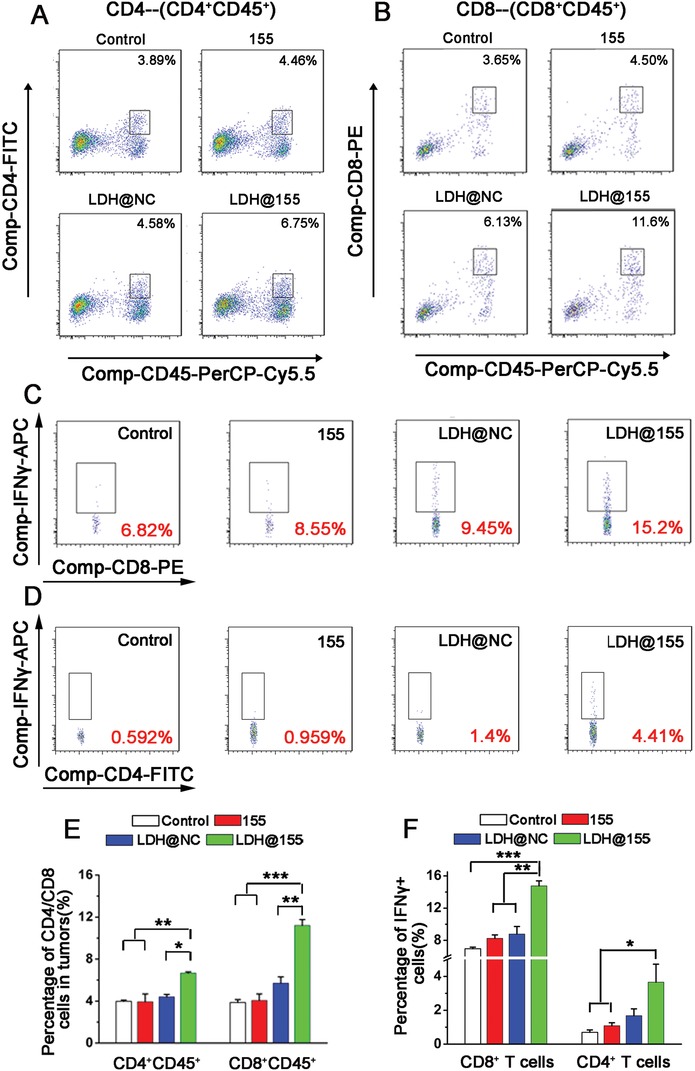
LDH@155 induced activated CD4^+^ and CD8^+^ T cells infiltration, CD4^+^IFNγ^+^ and CD8^+^IFNγ^+^ appreciation in TC‐1 environment. A,B) Flow cytometry plots of activated CD4^+^ and CD8^+^ T cells from TC‐1 tumors. C,D) Representative flow cytometry plots of IFNγ positive cells within the CD8^+^ T cells population (C) or CD4^+^ T cells population (D) of suspension cells from the TC‐1 tumors after different treatments. E) Quantification of CD4^+^ and CD8^+^ T cells of suspension cells from the TC‐1 tumors after different treatments. F) Percentage of IFNγ^+^CD8^+^ cells and IFNγ^+^CD4^+^ cells of suspension cells from the TC‐1 tumors after different treatments. Date are presented as mean + s.d. Statistical significance was calculated by using one‐way ANOVA. *p < 0.05; **p < 0.01; ***p < 0.001.

To confirm that LDH@155 exerted their antitumor roles not through killing tumor cells directly, but via remodeling immunoenvironment, we first observe the influence of LDH@155 on TC‐1 cells viability in vitro and its antitumor effect on immune‐deficient nude BALB/c mice in vivo. Expectedly, no obvious cytotoxicity and antitumor effect were found in LDH@155 treated TC‐1 in vitro (Figure S5, Supporting Information) and in immune‐deficient nude BALB/c mice i.t. injected with LDH@155 in vivo (Figure S6, Supporting Information), respectively. Next, we excised tumors from C57BL/6J at the end of the experiment and isolated the CD11b^+^ F4/80^+^ TAMs to observe the effect of LDH@155 on TAMs repolarization in vivo. As shown in Figure 4E, LDH@155 suppressed M2 phenotype switch in tumor environment evidenced by that M2 associated gene expression (Arg‐1) was inhibited and M1 gene expression (iNOS) was increased in TAMs. Considering M2 type TAMs could induce ITM by attracting MDSC infiltration and suppress T cells activation, we finally examined the immune cell composition of tumor sites after LDH@155 treatment. As shown in Figure 4, LDH@155 group significantly decreased the number of CD11b^+^ F4/80^+^ TAMs (Figure 4C,D) as well as CD11b^+^Gr1^+^ MDSCs (Figure 4F,G) in the TC‐1 tumors. Furthermore, expression level of immunosuppressive associated genes in MDSCs, including S100A8 and S100A9, were prominent decreased by LDH@155 treatment compared to control group (Figure 4H). By contrast, LDH@155 treatment markedly increased the number of CD8^+^ T and CD4^+^ T cells (**Figure** **5**A,B,E). IFN‐γ positive CD8^+^ T cells and CD4^+^ T cells in tumors were also increased by LDH@155, which represented LDH@155 activated more CD8^+^ T cells (Figure 5C,F) and CD4^+^ T cells (Figure 5D,F). Meanwhile, LDH and LDH@NC exhibited some degree of abilities of suppressing TAM and MDSCs infiltration compared to control.

Herein, LDH@155 played a vital role in reversing immunosuppressive tumor microenvironment through repolarizing the TAMs to M1 subtype indicated by infiltration deduction of TAMs and MDSCs, CD4^+^ T cells and CD8^+^ T cells activation and proliferation.

### 2.5 Biosafety of LDH@155 Nanoparticles In Vivo

Biosafety is also a primary concern of gene therapy based of nanoparticles, which is a critical factor for future clinical use.^45^ So we first evaluated the weight of mice of each treatment group during the whole treatment period. As shown in Figure S7 of the Supporting Information, all groups showed no significant difference during the experimental period, indicating good biocompatibility of nanoparticle‐based injection on mice. Then the mice were euthanized at the end of the experiment for blood biochemistry and histological analyses. The potential toxicity of LDH@155 in main organs (heart, liver, spleen, lung, kidney) was estimated via hematoxylin and eosin (H&E) staining (Figure S8A, Supporting Information). Treatment groups did not show any abnormalities in cellular morphology compared with control group. Meanwhile, serum alanine aminotransferase, aspartate aminotransferase, lactate dehydrogenase (LDH), blood urea nitrogen, and uric acid were measured to evaluated the renal and liver damages. As shown in Figure S8B,C of the Supporting Information, no obvious change was found among all groups. All these results indicated that introtumoral administration of LDH@155 was well biocompatible in vivo.

### 2.6 LDH@155 Nanoparticles Enhanced Therapeutic Effects of Anti‐PD‐1 Immunotherapy and Chemical Therapy

**Figure 6:**
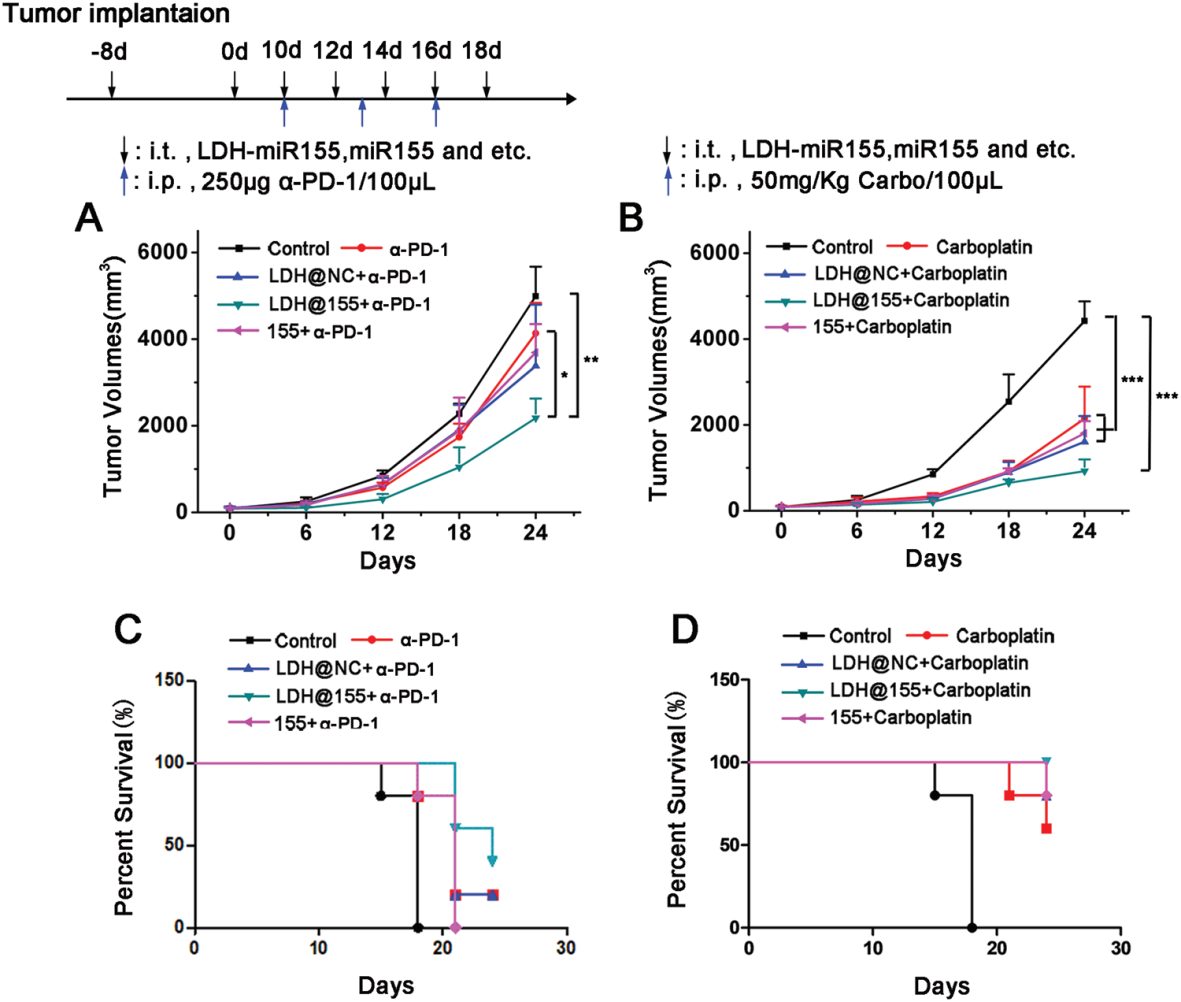
Enhancing therapeutic effects of chemo‐ and immunotherapy therapy by LDH@155. C57BL/6J tumor‐bearing mice (*n* = 5) received i.t. injection of PBS, LDH@NC, LDH@155, 155 at day 8 every other day for five times. Meanwhile, α‐PD‐1 antibody (250 µg per mouse) and carboplatin (50 mg kg^−1^) were i.p. at day 10 every 3 days for three times. A,B) Tumor growth curves of α‐PD‐1 treatment (A) and carboplatin treatment (B). C,D) Kaplan–Meier survival curves of α‐PD‐1 treatment (C) and carboplatin treatment (D). Date are presented as mean + s.d. Statistical significance was calculated by using one‐way ANOVA. *p < 0.05; **p < 0.01; ***p < 0.001.

Recent studies have shown that improving tumor immune environment may facilitate responses to chemo‐ and immunotherapy in solid tumor.^46^ In addition, excessive tumor infiltration of myeloid cells may further blunt the efficacy of both chemotherapy and immunotherapy. Given that LDH@155 reduced TAMs and MDSCs infiltration and increased T cells activation in tumor, we sought to test the ability of LDH@155 to improve chemo‐ and immunotherapeutic efficacy. We first evaluated combinations of checkpoint immunotherapies with LDH@155 in syngeneic TC‐1 models. As shown in **Figure** **6**A,C, LDH@155 treatment promoted the responsiveness to a PD‐1 antagonist (α‐PD‐1) in TC‐1 model, as seen by reduced tumor burden and improved overall survival.

To assess the ability of LDH@155 to improve efficiency of chemotherapy, we next treated mice bearing TC‐1 tumors with either vehicles or high‐dose carboplatin (50 mg kg^−1^ of body weight, intraperitoneal (i.p.)) in the presence or absence of LDH@155. We found that compared with using carboplatin alone, combined therapy of LDH@155 and carboplatin showed a more excellent ability in TC‐1 tumor recession and prolonged overall survival (Figure 6B,D).

Taken together, these data indicated that LDH@155 can enhance solid tumors responsive to chemo‐ and immunotherapy through altering the ITM to facilitate CD4^+^ and CD8^+^ activation and infiltration and diminish the M2 type TAMs and MDSCs in solid tumor, which could provide clinical significance for combined therapy. Combining checkpoint inhibitors with other immunotherapy agents has demonstrated synergistic effects in cancer therapy.^47^, ^48^ However, the combination of multiple therapeutics frequently appears to induce stronger toxicity.^49^ Furthermore, since the drugs used in combination have widely different properties including pharmacokinetics, biodistribution, and mechanisms of actions, it may be difficult to optimize the synergistic effect.^50^ In this study, we found that LDH@155 could augment the therapeutic efficiency in combination immunotherapy by overcoming biological barriers and delivering specifically. Thus, combinations of checkpoint immunotherapies with LDH@155 may providing a new idea for combined immunotherapy for cancer patients.

### 2.7 LDH@155 Nanoparticles Repolarized TAMs to M1 Subtype in STAT3, ERK1/2, and NF‐κB Dependent Manner

**Figure 7:**
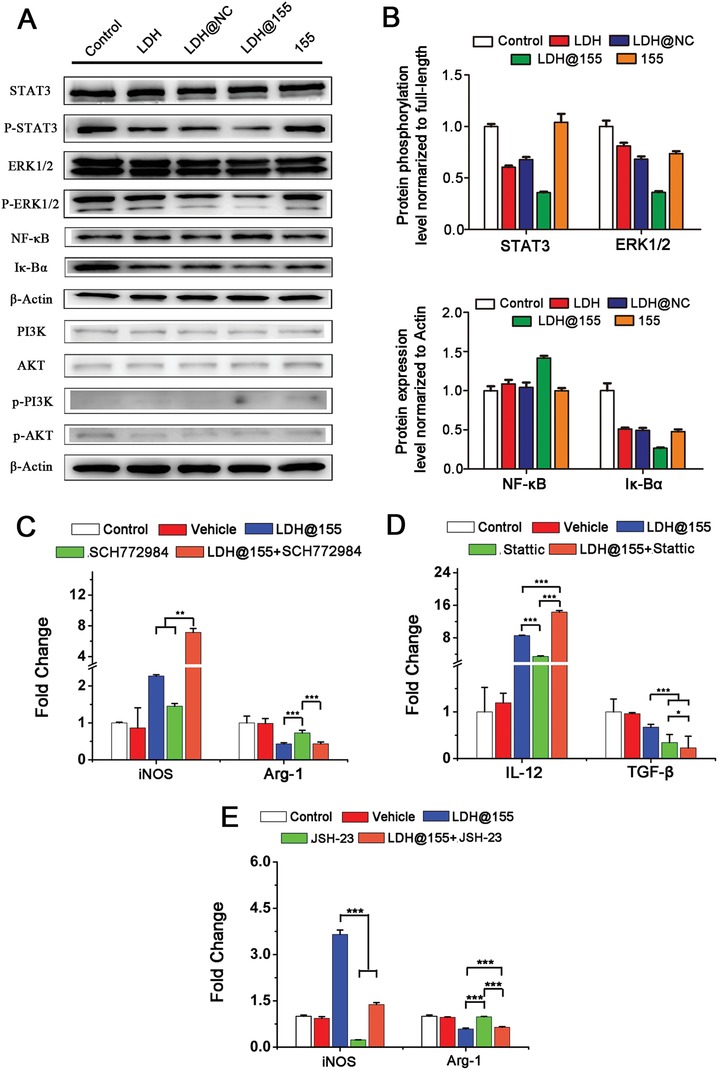
The signaling pathways and mechanism of LDH@155 on repolarize M2 macrophage to M1 macrophage. A) Western Blot assay of the selected proteins for the determination of phosphorylation and full‐length antibodies array. B) Quantitative analysis of the relative protein expression level. C) mRNA expression level of M1 maker (iNOS) and M2 marker (Arg‐1) with treatment of ERK1/2 inhibitor (SCH772984, 10^−5^ m) for 24 h. D) mRNA expression level of M1 maker (IL‐12) and M2 marker (TGF‐β) with treatment of STAT3 inhibitor (Stattic, 2 × 10^−6^ m) for 24 h. E) mRNA expression level of M1 maker (iNOS) and M2 marker (Arg‐1) with treatment of NF‐κB inhibitor (JSH‐23, 10^−5^ m) for 24 h. Date are presented as mean + s.d. Statistical significance was calculated by using one‐way ANOVA. *p < 0.05; **p < 0.01; ***p < 0.001.

To further illuminate the molecular mechanisms involved in LDH@155 induced TAMs repolarization, downstream signal pathways including the ERK1/2, NF‐κB (p‐65), AKT, PI3K, and STAT3, were investigated. First, the expression levels of all these proteins were determined by Western blot analysis. As shown in **Figure** **7**A,B, the bands of p‐STAT3, p‐ERK1/2, and Iκ‐Bα got weaken under LDH@155 treatment compared with control. Whereas the band of p‐PI3K and p‐AKT showed no obvious change between LDH@155 treatment and control. These results suggested that STAT3, ERK1/2, and NF‐κB pathway might involve in LDH@155 induced TAMs repolarization (Figure 7A). To further confirm this result, ERK inhibitor (SCH772984) and STAT inhibitor (Stattic) or NF‐κB inhibitor (JSH‐23) were used to cotreat TAMs with LDH@155 and then the expression of M1 and M2 markers were measured. From the result in Figure 7C, either ERKi (SCH772984) treatment or coprocessing of Stattic and LDH@155 exhibited similar effects with LDH@155 which consequently led to M1 polarization. In addition, Stattic showed consistent results in accordant with SCH772984 (Figure 7D). Whereas NF‐κBi (JSH‐23) treatment suppressed M1 subtype switch induced by LDH@155 (Figure 7E). Hence, all these results vertified p‐STAT3, p‐ERK1/2, and NF‐κB may be critical pathways participating in TAMs repolarization with the LDH@155 treatment.

Recently, it is well known that STAT3, ERK1/2, and NF‐κB pathways were involved in multiple biological processes including cell proliferation, differentiation, apoptosis, and immune response.^51^, ^52^, ^53^ Previous studies have shown that SOCS1‐STAT3‐NF‐κB and ERK1/2 signaling pathways participated in macrophages activation induced by miR155.^54^ Consistent with the above research, our present study also revealed that STAT3 and ERK1/2 phosphorylated signal pathways were remarkable inhibited by LDH@155 in TAMs repolarization. Furthermore, our previous study showed NF‐κB signaling pathway played a vital role in MDDCs activation induced by LDHs.^39^ Here, we also confirmed that NF‐κB pathway also was involved in repolarization of macrophages by LDHs@miR155.

## 3 Conclusion

**Figure 8:**
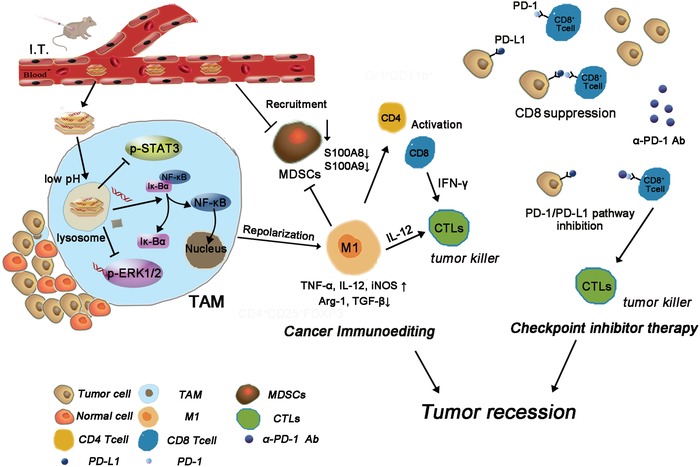
Schematic diagram of checkpoint inhibitor therapy combined with cancer immunoediting through LDH@155.

In summary, we have presented an acidity sensitive LDH@155 nanoparticles for enhanced chemo and immunotherapy by strongly reversing the ITM. Our results showed that LDH@155 displayed positive surface charge and was passively swallowed by tumor associated macrophages. LDH@155 could effectively release miR155 in the intracellular acidity microenvironment. Importantly, we demonstrated that LDH@155 synergistically skewed TAMs to M1 subtype in STAT3, ERK1/2, and NF‐κB dependent manner, which led to induce higher T‐cell activation and inhibit MDSCs infiltration in tumor environment (**Figure** **8**). Furthermore, LDH@155 could improve chemo and immunotherapy and was significantly more effective than free antibody therapeutics or single drug therapeutic. Given the merits of TAMs uptake, pH‐sensitive miR release inside the lysosome, as well as the robust remolding the ITM, our study suggested that LDH@155 is of potent potential for improved immunotherapy.

## 4 Experimental Section

*Materials*: Mg (NO_3_)_2 _· 6H_2_O, Al (NO_3_)_3_ · 9H_2_O, and NaOH were obtained from Sinopharm Chemical Reagent Co, Ltd. (Shanghai, China). 3‐(4,5‐dimethylthiazol‐2‐yl)‐2,5‐diphenyl tetrazolium bromide (MTT) was purchased from Sigma‐Aldrich (MO, USA). Dulbecco's modified Eagle's Medium (DMEM) (high glucose) medium, RPMI1640 basic medium, fetal bovine serum, trypsin, penicillin, and streptomycin were purchased from Thermo Fisher Scientific (MA, USA). LysoTracker green, DAPI were purchased from Keygen (Nanjing, China). Small molecular inhibitors including SCH772984, Stattic, and JSH‐23 were obtained from Apexbio (Shanghai, China). MicroRNA mimics and control miR were synthesized by Ribio (Guangzhou, China). Fluorescent‐labeled monoclonal antibodies were purchased from eBioscience (CA, USA). M‐CSF and IL‐2 recombinant proteins were purchased from PeproTech (NJ, USA). α‐PD‐1 antibody and carboplatin were supplied from BioXcell (NH, USA) and Mellon Biology (Dalian, China), respectively.

*Cells and Animals*: TC‐1 murine cervical cells and RAW264.7 murine macrophage‐like cells were obtained from Chinese Academy of Sciences (Shanghai, China). Cell lines were cultured in DMEM (high glucose) medium supplemented with 10% fetal bovine serum (FBS), and penicillin/streptomycin (50 units mL^−1^) at 37 °C and 5% CO_2_ in a humidified incubator. TAMs were generated as the laboratory existing method. Briefly, cells extracted from abdomen of 6–8 weeks C57BL/6J female mice were diluted at a concentration of 10^6^ cells mL^−1^ and cultured in RPMI 1640 medium supplemented with 10% FBS, penicillin/streptomycin (50 units mL^−1^), and recombinant M‐CSF (20 ng mL^−1^). After placed in a humidified incubator (37 °C, 5% CO_2_) overnight, all of the loosely adherent cells were discarded once macrophages were adhered to cell plate and fresh medium supplemented with M‐CSF (20 ng mL^−1^) and 30% TC‐1 culture supernatant was added to maintain cultivation for another 3 days. Then TAMs were induced successfully and used for subsequent experiments in vitro.

*Animals*: C57BL/6J mice (6–8 weeks old, female) and BALB/c nude mice (4–6 weeks old, female) were purchased from the Shanghai Laboratory Animal Co. Ltd. (Shanghai, China) and housed in the pathogen‐free animal facility of Tongji University. All animal experimental protocols were approved by Institutional Laboratory Animal Resources at University.

*Synthesis and Characterization of LDH@miR NPs*: The chemical synthesis of Mg–Al LDH nanoparticles were performed by coprecipitation method according with previous report with slight revision.^39^ Then colloidally stable LDH NPs were formulated as uniform liquid phase via ultrasound for 1 h. Lyophilized powder of miR was resolved by RNA‐free water. Then LDH@miR nanoparticles were prepared by shaking the mixed solution of LDH NPs and miR (LDH:miR = 20:1, w/w) at 500 rpm at 4 °C for 30 min. LDH@NC (negative control miRNA) was obtained by the same method. Dynamic light scattering (DLS) experiments were performed by Zetasizer NanoZS Instruments and zeta potential values were estimated on the same equipment at 25 °C. The samples were dispersed in PBS (pH 7.4) under ultrasound for 2 h, and then measured at room temperature. Powdered sample XRD patterns were recorded on a Rigaku Diffractomer Model Miniflex using Cu Kα radiation. TEM imaging was obtained on a transmission electron microscope (JEOL, Tokyo, Japan) at 100 kV. SEM imaging was received by a scanning transmission electron microscope (JEM‐2100F, Japan).

*Loading Ratio, Acid Release of miR*: For loading ratio assay, different samples were subjected to 1.5% agarose gel electrophoresis in TAE buffer containing GelRed (Beyotime, China) at 100 V for 20 min. For pH‐sensitive release assay, samples were treated with PBS solutions at different pH values of 7.4, 6.5, 5.5, and 4.5 for 1 h, respectively. And at constant pH value of 6.0, the ability of pH‐sensitive release was also verified with the extension of the time at 20 min, 40 min, 1 h, 2 h, and 4 h. 0.4 µg of miR was run as positive control and visualized using a Molecular Imaging System (Tanon, China).

*Cellular Uptake and Location Assay of miR by Macrophages In Vitro*: RAW264.7 cells were seeded in glass bottom dishes at a density of 10^5^ cells per dish for 24 h and treated with 50 × 10^−9^ m miR‐Cy5 or LDH@miR‐Cy5 (equivalent miR‐Cy5 concentration: 50 × 10^−9^ m) at interval time or under different pH atmosphere. The uptake of miR was evaluated by confocal laser scanning microscopy (TCS SP5II, Leica, Germany). To determine cellular location of LDH@miR, cells were incubated with free miR‐Cy5 or LDH@miR‐Cy5 for 3 h and stained with Lysotracker Green (Ex: 443 nm, Em: 505 nm) and DAPI (Ex: 358 nm, Em: 461 nm). Then the cellular localization was recorded by a confocal laser scanning microscopy (Leica TCS SP5 II, Germany).

*In Vitro MDSC Induction*: TAMs had been reported on influence of the MDSC formation so the effect of TAMs repolarization on MDSCs formation was intended to evaluate.^7^ TAMs were induced and plated at substratum of 24 well Transwell plate (Corning, USA). Then the cells were treated with LDH, LDH@NC, LDH@155, free miR155 at the miR concentration of 100 × 10^−9^ m with complete medium containing 30% TC‐1 cell supernatant. Bone marrow cells were isolated from tibia of 6–8 weeks C57BL/6J female mice then residual organizations were filtered out using a 70 µm cell strainer (BD Bioscience, USA). Cells were diluted to 10^6^ cells/300 µL and plated in upper panel at the same time with RPMI‐1640 medium supplemented with 10% FBS and 50 units mL^−1^ streptomycin/penicillin. After cocultured for 3 days, the percentage of MDSC (Gr1^+^CD11b^+^) in cell suspensions of bone marrow cells was detected by flow cytometry (BD FACS Verse, USA).

*In Vitro CD4^+^ and CD8^+^ T Cell Priming Assay*: TAMs had been known to inhibit activation of CD8^+^ T cells through secreting cytokines such as IL‐10, TGF‐β.^55^ Herein, the influence of TAMs repolarization on IFN‐γ secretion, which was used to quantify CD8^+^ T cell activation, was intended to investigate. Lymphocytes cells were obtained from lymph grands of 6–8 weeks C57BL/6J female mice. Briefly, lymph grands were plated in 6‐well cell plate and extruded with injection syringe. After filtered with 70 µm cell strainer (BD Bioscience, USA), cells were diluted to 5 × 10^6^ mL^−1^ and plated in U‐type of 96 well with complete RPMI 1640 medium (10% FBS, 50 units mL^−1^ streptomycin/penicillin and 10 ng mL^−1^ IL‐2). Half volume of medium was replaced by TAMs cultural supernatant treated with LDH, LDH@NC, LDH@155, free miR155 for 48 h at the miR concentration of 100 × 10^−9^ m. Then CD3, CD28 antibody (1:1000 dilutions) were added to stimulate T‐lymphocyte activation. BrefeldinA (eBioscience, CA, USA) was added at the final concentration of 3 µg mL^−1^ to prevent extracellular secretion at the third day. Then percentage of IFN‐γ positive cells in CD8^+^ T cells or CD4^+^ T cells was labeled via intracellular staining and detected by flow cytometry.

*Western Blot Assay*: Anti‐NF‐κB, anti‐Iκ‐Bα, anti‐p‐STAT3, and anti‐STAT3, anti‐p‐ERK1/2 and anti‐ERK1/2, anti‐AKT and anti‐p‐AKT, anti‐p‐PI3K, and anti‐PI3K, β‐Actin antibodies were purchased from Cell Signaling Technology (MA, USA). TAMs were treated with LDH, LDH@NC, LDH@155, free miR155 with complete medium containing 30% TC‐1 cell supernatant for 48 h. Then the proteins were obtained by total protein extraction kit (KeyGen Biotech, Nanjing, China) and the protein concentration was calculated by the BCA protein assay (KeyGen Biotech, Nanjing, China). An aliquot of 50 µg of total extract was mixed with a 5× protein‐loading buffer (KeyGen Biotech, Nanjing, China) and boiled for 5 min before loading onto an 12% SDS‐PAGE gel. After electrophoresis, proteins were transferred into PVDF membranes (EMD Millipore, Billerica, MA, USA) at 300 mA for 100 min in an ice bath. Then nonspecific binding was blocked by incubation with 5% BSA solution containing 0.1% Tween‐20 (TBST) for 30–60 min at room temperature. The blocked PVDF membranes were incubated with primary antibodies (1:1000 dilutions) at 4 °C overnight and washed three times with TBST. After incubated with HRP‐conjugated secondary antibodies (1:5000 dilutions), the bands were visualized using ImageQuant LAS4000 mini (GE Healthcare Life Science, USA).

*Quantitative Real Time PCR (qRT‐PCR) Assay*: Total RNA was extracted using Trizol reagent (Takara, Dalian, China). Then cDNA was obtained by reverse transcription using a cDNA transcription kit (Takara, Dalian, China) according to manufacturer's instructions. qRT‐PCR was performed using SYBR Green mix from Takara on Light Cycler 96 (Roche, USA). For miR155, U6 was used as internal control to normalize the relative expression. For mouse Arg‐1, TGF‐β, iNOS, IL‐12, TNF‐α, S100‐A8, S100‐A9, and GAPDH were selected as internal control. Primers sequences were shown in Table S1 of the Supporting Information.

*In Vivo TAM‐Targeting and Intratumoral Metabolism of LDH@miR*: TC‐1 cells (10^6^/100 µL) were injected subcutaneously into the right flank of the 6–8 weeks old female C57BL/6J mice. When the tumors reached about 500–600 mm^3^, tumor‐bearing mice were i.t. injected with LDH@miR‐Cy5 and miR‐Cy5 with the dosage of 500 pmol per mouse per time. Then tumor fluorescence images were recorded from 30 min to 24 h using the NightOwl LB983 in vivo imaging system (NightOwl, USA). To further determine whether LDH@miR‐Cy5 could realize CD11b^+^ cells (mainly TAMs) passively targeting, tumor tissues were harvested at 48 h and digested with the mixture of collagenase type I (0.05 mg mL^−1^, Sigma), collagenase type IV (0.05 mg mL^−1^, Sigma) and Dnase I as previously described. The cell suspension was labeled with mouse anti‐CD11b antibody and the cellular uptake of miR was measured via the flow cytometry (BD FACS Verse, USA).

*Tumor Volume Evaluation*: TC‐1 cells (5 × 10^5^ cells) were suspended in 100 µL PBS and inoculated subcutaneously into the right flank of C57BL/6J mice. About 7 days later, when the tumors reached about 100–150 mm^3^, tumor‐bearing mice were i.t. injected with different treatments every other day for five times. For anti‐PD‐1 and carboplatin synergistic treatment, α‐PD‐1 antibodies (250 µg per mouse) and carboplatin (50 mg Kg^−1^) were i.p. injected every 3 days for three times combined with different NPs i.t. injection as shown in Figure 6. The dosage of LDH NPs and miR were 160 µg and 500 pmol per mouse per time, respectively. The volumes of the tumors were measured every 3 days using a digital caliper and calculated as the formula: Tumor volumes = (length*width^2^)/2.

*Immunoediting of TC‐1 Tumor Environment*: Two days after the last injection, the tumors were digested as above described. The partial obtained cell suspension was labeled with mouse antibodies against CD4‐FITC, CD8‐PE, CD11b‐FITC, F4/80‐APC, Gr‐1‐PE to identify activated CD4 cells (CD4^+^CD45^+^) and CD8 cells (CD8^+^CD45^+^), TAMs (F4/80^+^CD11b^+^), and MDSCs (CD11b^+^Gr‐1^+^), respectively. To evaluate percentage of activated CD8^+^ T (IFN‐γ^+^CD8^+^) or activated CD4^+^ T cells (IFN‐γ^+^CD4^+^) in tumor environment, single cell suspension was plated in U‐type 96 well cell plate and treated with IL‐2 (20 ng mL^−1^), CD3 and CD28 antibodies (1:1000). Cells were cultured in cell incubator overnight. Then BrefeldingA solution was added each well at the final concentration about 3 µg mL^−1^. After 4–6 h, Intracellular Fixation & Permeabilization Buffer Set Kit (eBioscience, USA) was used to label IFN‐γ in cytoplasm. All immune cell populations were analyzed using flow cytometry. TAMs (F4/80^+^CD11b^+^) and MDSCs (CD11b^+^Gr‐1^+^) were sorted from tumor cell suspension by flow cytometer (BD FACS AriaII, USA). Expression level of M1 markers (iNOS), M2 markers (Arg‐1) on TAMs and S100A8 and S100A9 of MDSCs were evaluated through qRT‐PCR.

*In Vivo Biosafety Evaluation*: At 48 h after the last injection, mice were euthanized and the blood samples were collected. Samples were sent to Center for Drug Safety Evaluation and Research, Shanghai University of T.C.M. and analyzed by a HITACHI 7080 Automatic Biochemical Analyzer. To evaluate histopathological damage, major organs (heart, liver, spleen, lung, and kidney) were taken out and fixed in 4% paraformaldehyde overnight, then embedded in paraffin and cut into 5 mm sections for H&E staining according to standard procedures and the histology of different organs were using a light microscope (Olympus Corporation, Tokyo, Japan).

*Data Analysis*: Data analyses were performed by Graph Pad software for windows. Significant difference was analyzed using Student's *t*‐test and one‐way ANOVA. Data were shown as mean ± s.d. And the criterion for differences (*p < 0.05; **p < 0.01; ***p < 0.001) were considered statistically significant.

## Conflict of Interest

The authors declare no conflict of interest.

## Supporting information

Supplementary
