# Supplementary material for: Synergetic Functional Nanocomposites Enhance Immunotherapy in Solid Tumors by Remodeling the Immunoenvironment
Source: Adv Sci (Weinh). 2019 Feb 16;6(8):1802012. doi: 10.1002/advs.201802012 (PMC6469336; doi:10.1002/advs.201802012)
Supplement: Supplementary file 1 — Supplementary [file ADVS-6-1802012-s001.pdf]

Copyright WILEY-VCH Verlag GmbH & Co. KGaA, 69469 Weinheim, Germany, 2019.

ADVANCED SCIENCE
Open Access

Supporting Information

for *Adv. Sci.,* DOI: 10.1002/advs.201802012

Synergetic Functional Nanocomposites Enhance Immunotherapy in Solid Tumors by Remodeling the Immunoenvironment

Linnan Yang, Jing Sun, Qiang Liu, Rongrong Zhu, Qiannan Yang, Jiahui Hua, Longpo Zheng, Kun Li, Shilong Wang, and Ang Li**

Supporting Information

Synergetic Functional Nanocomposites Enhance Immunotherapy in Solid Tumor via Remodeling Immunoenvironment

Linnan Yang#, Jing Sun#, Qiang Liu, Rongrong Zhu, Qiannan Yang, Jiahui Hua, Longpo Zheng, Kun Li, Shilong Wang, Ang Li*.*

L. Yang, J. Sun, Q Liu, Prof. R. Zhu, Q. Yang, J. Hua, K. Li, Prof. S. Wang, Prof. A. Li,

Research Center for Translational Medicine at East Hospital, Shanghai First Maternity & Infant Health Hospital, School of Life Science and Technology, Tongji University, Shanghai 200092, People's Republic of China

* Corresponding Author:

Shi-Long Wang, Email: wsl@tongji.edu.cn

Ang Li, Email: liang@tongji.edu.cn

L. Zheng

Shanghai Tenth People's Hospital, School of Medicine, Tongji University, Shanghai 200092, People's Republic of China

# Both authors contributed equally to this work

Cytotoxic effect of LDH@155 NPs: The cytotoxic effect of nanoparticles was evaluated by MTT assay as previously reported. Briefly, TAMs (6×10^3 cells per well) and TC-1 murine cervical cells (5×10^3 cells per well) were plated at 96-well cell plates and treated with free miR155, LDH NPs and LDH@155 at the miR concentration from 0 to 200×10^{-9} M for 24 h and 48 h, respectively. Then MTT (5 mg/ml) was added and cells were further incubated for another 4 h. 150μl DMSO was added after the cultural supernatant was removed. Finally, OD values was measured by a microplate reader (ELX800 UV, BIO-TEK, USA) at 492 nm.

Anti-tumor effect on nude BALB/c mice: TC-1 cells (2×10^6) were suspended in 100 ul PBS and inoculated subcutaneously into the right flank of nude BALB/c mice. About seven days later, when the tumors reached about 100 mm^3, tumor-bearing mice were i.t. injected with different treatments every other day for 4 times. The dosage of LDH NPs and miR was 160 μg and 500 pmol/mouse/time, repectively. The volums of the tumors were measured every two days using a digital caliper and calculated as the formula: Tumor volumes = $(length*width^2)/2$. The body weight was weightd by an electronic balance.

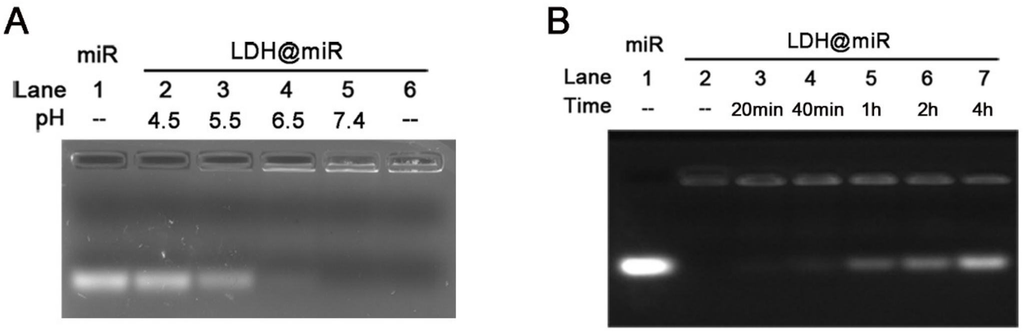

Figure S1. (A) Acid release of miR at different pH values. Lane 1: free miR; Lane 2-5: shaking of LDH@miR at pH value of 4.5, 5.5, 6.5, 7.4, respectively. Lane6 ：LDH@miR. (B) Acid release of miR at different time point at constant pH value of 6.0. Lane1: free miR; Lane2: LDH@miR; Lane 3-7: shaking of LDH@miR at pH value of 20 min, 40 min, 1 h, 2 h, and 4 h, respectively.

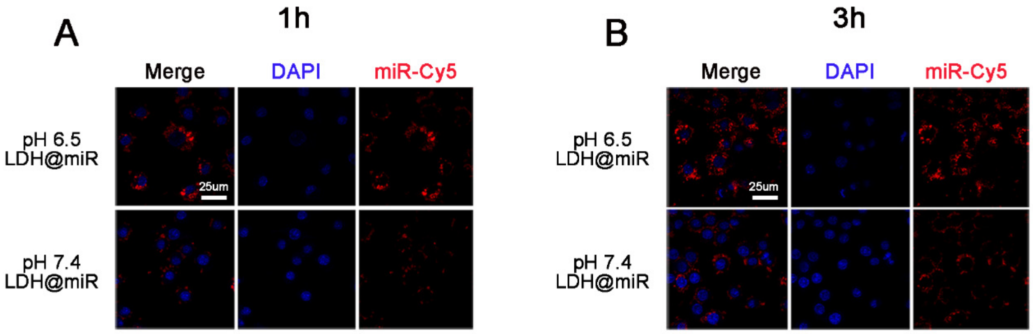

Figure S2. Fluorescence confocal images of LDH@miR-Cy5 uptake by RAW264.7 at pH 7.4 and pH 6.5 for (A) 1 h and (B) 3 h, respectively. Bar = 25 μm.

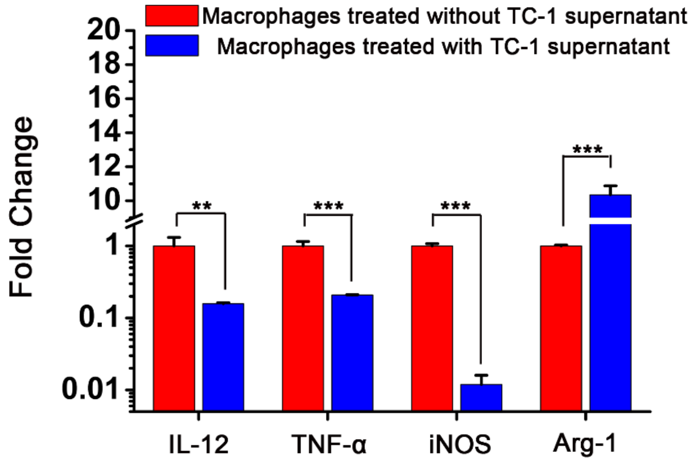

Figure S3. mRNA expression levels of M1, M2 markers (IL-12, TNF-α, iNOS, Arg-1) in macrophages treated with/without TC-1 supernatant were detected via qRT-PCR. Date are presented as mean + s.d. Statistical significance was calculated by using one-way ANOVA. * $p < 0.05$; ** $p < 0.01$; *** $p < 0.001$.

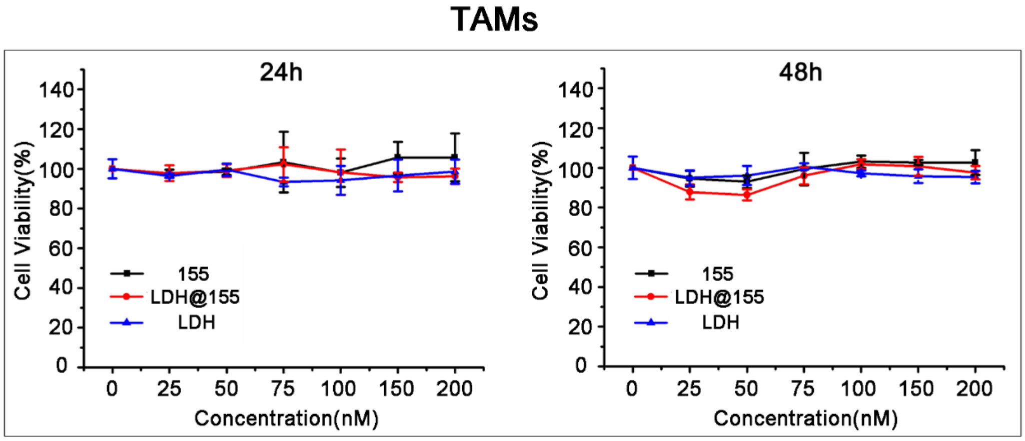

Figure S4. Cytotoxicity assay of LDH NPs, LDH@155, free miR155 on TAMs for 24 h and 48 h, respectively. Date are presented as mean ± s.d.

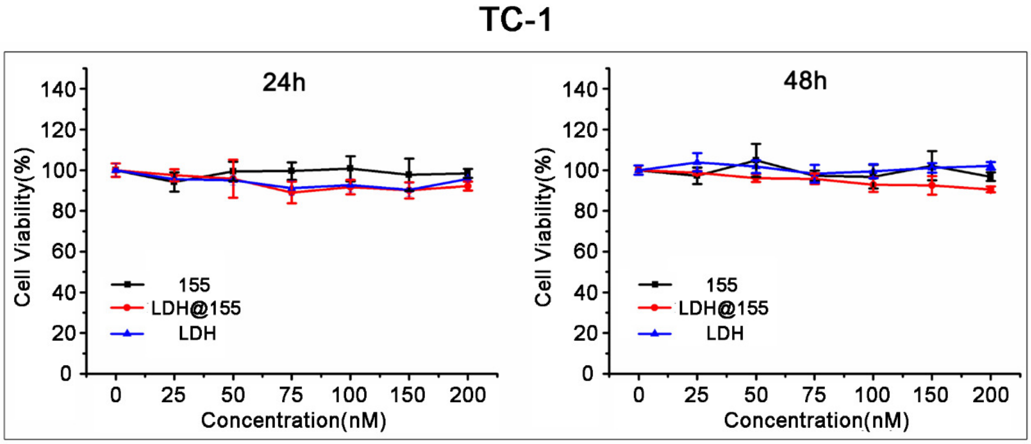

Figure S5. Cytotoxicity assay of LDH NPs, LDH@155, free miR155 on TC-1 cells for 24 h and 48 h, respectively. Date are presented as mean ± s.d.

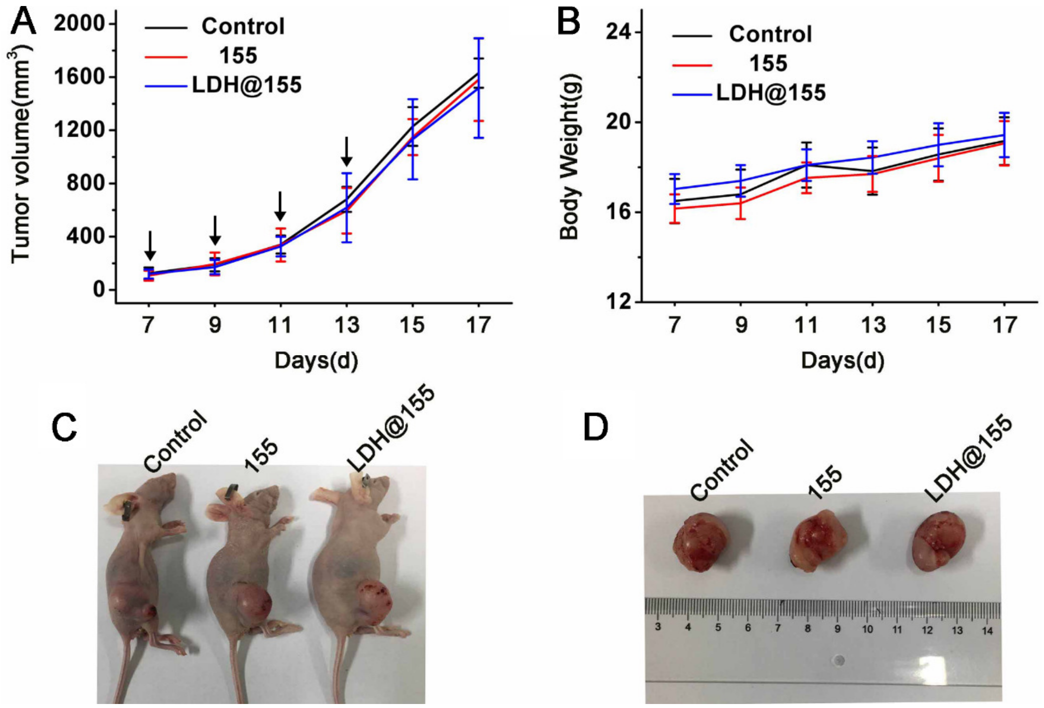

Figure S6. Anti-tumor ability of free miR155 and LDH@155 on nude BALBc mice (n = 4). (A) The tumor area was monitored every 2 days. (B) Body weight was measured every 2 days. (C). Digital images of tumor-bearing mice on day 17. (D)Side-by-side comparison of tumors on day 17 after tumor extracted from body. Date are presented as mean ± s.d.

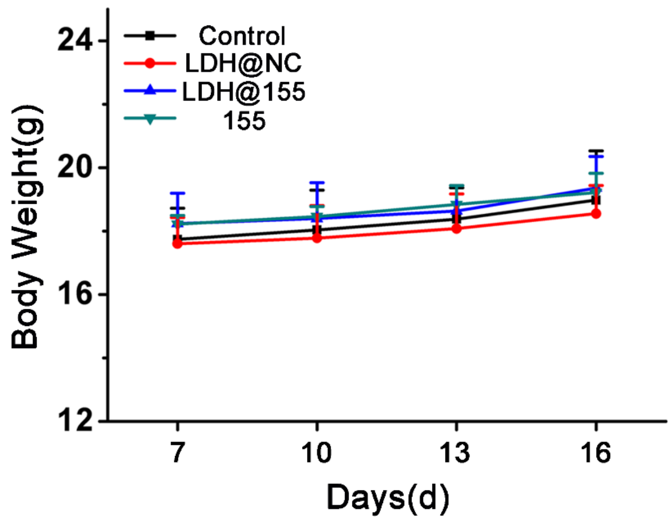

Figure S7. Body weight of each group was monitored every 3 days.

Date are presented as mean + s.d.

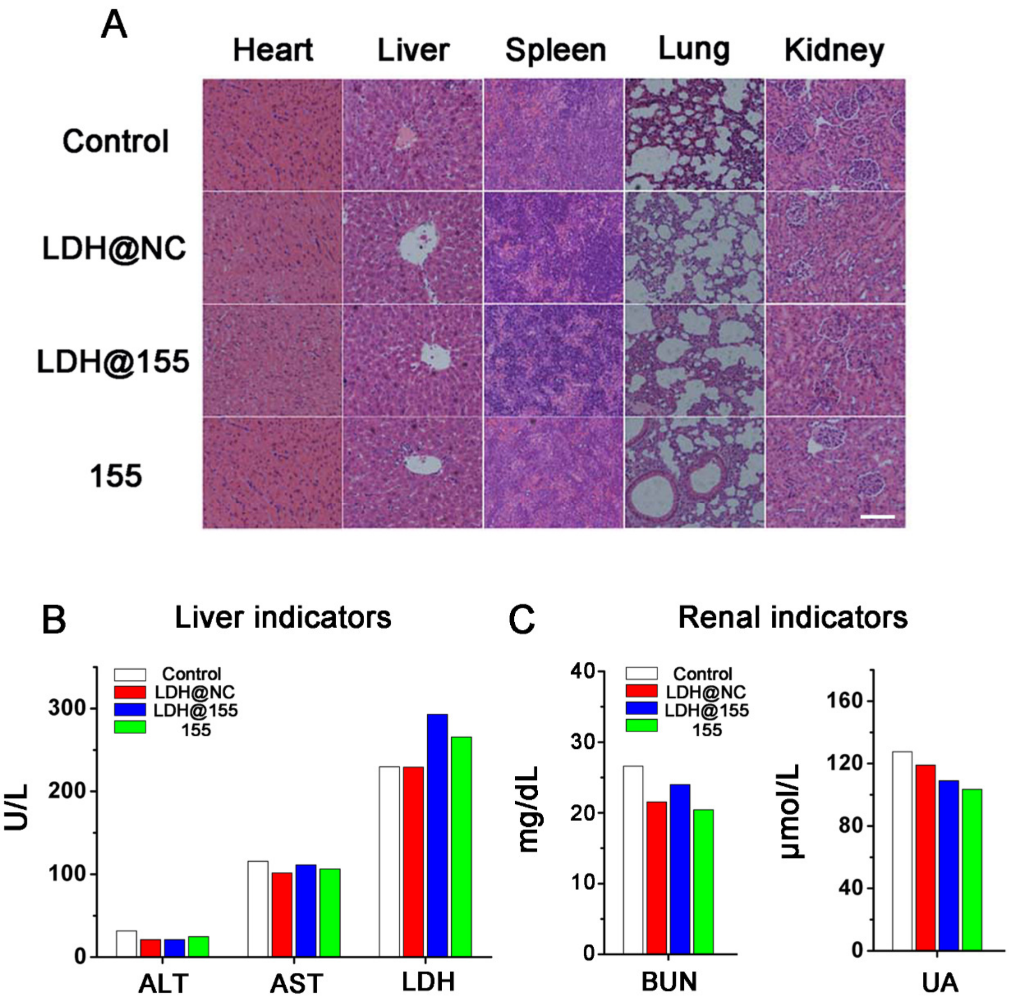

Figure S8. Biosafety evaluation of LDH@155 *in vivo*. (A) H&E staining of mouse organs (hearts, livers, spleens, lungs, and kidneys) at the end of experiments. Bar = 100 μm. (B) Serum levels of ALT, AST, LDH (liver functions). (C) Serum levels of BUN, UA (renal functions) at 48 h after last treatment.

Table S1. Primer lists

	F	R
**Arg-1**	CTCCAAGCCAAAGTCCTTAGAG	AGGAGCTGTCATTAGGGACATC
**TGF-β**	TGCTGCTTTCTCCCTCAACCT	CACTGCTTCCCGAATGTCTGA
**TNF-α**	CCCTCACACTCAGATCATCTTCT	GCTACGACGTGGGCTACAG
**iNOS**	ATCTTTGCCACCAAGATGGCCTGG	TTCCTGTGCTGTGCTACAGTTCCG
**IL-12-p70**	CCAAATTACTCCGGACGGTTCAC	CAGACAGAGACGCCATTCCACAT
**S100A8**	CTACTGAGTGTCCTCAGTTTGTGCAG	CCATCGCAAGGAACTCCTCGAAG
**S100A9**	GCGCAGCATAACCACCATCATCG	GCCAACTGTGCTTCCACCATTTG
**GAPDH**	GTGTTCCTACCCCCAATGTGT	ATTGTCATACCAGGAAATGAGCTT
